# Asperlin Inhibits LPS-Evoked Foam Cell Formation and Prevents Atherosclerosis in ApoE^−/−^ Mice

**DOI:** 10.3390/md15110358

**Published:** 2017-11-14

**Authors:** Yue Zhou, Ran Chen, Dong Liu, Chongming Wu, Peng Guo, Wenhan Lin

**Affiliations:** 1 Pharmacology and Toxicology Research Center, Institute of Medicinal Plant Development, Chinese Academy of Medical Sciences, Peking Union Medical College, Beijing 100193, China; xiaoyuzhou5213@sina.com (Y.Z.); cran_lxj@sina.com (R.C.); 2 State Key Laboratory of Natural and Biomimetic Drugs, Peking University, Beijing 100191, China; liudong_1982@126.com

**Keywords:** asperlin, inflammation, macrophage, M1/M2 polarization, atherosclerosis, foam cell

## Abstract

Asperlin is a marine-derived natural product with antifungal and anti-inflammatory activities in vitro. In the present study, we isolated asperlin from a marine *Aspergillus versicolor* LZD4403 fungus and investigated its anti-atherosclerotic effects in vitro and in vivo. Asperlin significantly inhibited lipopolysaccharides (LPS)- but not oxidated low-density lipoprotein (oxLDL)-evoked foam cell formation and promoted cholesterol efflux in RAW264.7 macrophages. Supplementation with asperlin also suppressed LPS-elicited production of pro-inflammatory factors in RAW264.7 macrophages, decreased the expression levels of iNOS, IL-1β and TNFα, and increased the expression of IL-10 and IL-4, indicating a remarkable shift in M1/M2 macrophages polarization. In vivo experiments in high-fat diet (HFD)-fed ApoE^−/−^ mice showed that oral administration of asperlin for 12 weeks remarkably suppressed atherosclerotic plaque formation in the aorta, as revealed by the reduced aortic dilatation and decreased atherosclerotic lesion area. Asperlin also decreased serum levels of pro-inflammatory factors but showed little impact on blood lipids in ApoE^−/−^ atherosclerotic mice. These results suggested that asperlin is adequate to prevent atherosclerosis in vivo. It may exert atheroprotective function through suppressing inflammation rather than ameliorating dyslipidemia.

## 1. Introduction

Atherosclerosis (AS), a chronic disease characterized by lipid accumulation and chronic inflammation of the arterial wall, is the pathologic basis of coronary heart disease, cerebrovascular disease and thromboembolic disease [1]. Nowadays, AS and its main complications constitute the first cause of death worldwide. Macrophage-derived foam cells have long been recognized as a hallmark of AS [2]. When cells such as macrophages and endothelial cells assimilate modified lipids including oxidized LDL (oxLDL) without restraint, foam cells are formed. Many regulatory proteins, such as peroxisome proliferator-activated receptor γ (PPARγ), liver X receptor-α (LXRα), ATP-binding cassette transporter A1 (ABCA1) and ABCG1, are known to play key roles in promoting cholesterol efflux from oxLDL-loaded macrophages [3,4]. Promoting cholesterol efflux by upregulation of PPARγ/LXRα/ABCA1 provides an efficient approach to prevent macrophage foam cell formation and atherosclerotic plaque occurrence. 

In the process of atherosclerosis, foam cells gradually converged on the artery walls, progressively leading to an oxidative lesion and the formation of thrombogenicity [5]. Macrophages further promote lesion inflammation through secretion of cytokines. Cytokines such as interleukin-1β (IL-1β), tumor necrosis factor α (TNFα) and interferon-γ (IFNγ) promote the expression of pro-inflammatory cytokines and chemokines and disturb lipid metabolism in macrophages, which promotes plaque rupture in blood vessels [6]. In contrast, other cytokines such as IL-10 exert anti-inflammatory properties by reducing the expression of pro-inflammatory cytokines and normalizing lipid metabolism in macrophages [7]. Similarly, IL-4 is also shown to possess an anti-atherosclerotic function [8]. 

A great deal of evidence has demonstrated that macrophages can adopt different phenotypes in response to a particular stimulus [9,10,11]. Macrophages stimulated with interferon γ (IFNγ) and lipopolysaccharides (LPS) adopt a M1 phenotype which release cytokines that inhibit the proliferation of surrounding cells and damage contiguous tissue [11]. Conversely, macrophages stimulated with IL-4 and IL-13 adopt an anti-inflammatory M2 phenotype and release cytokines that promote the proliferation of contiguous cells and tissue repair [9]. The M1/M2 classification is commonly used to define the macrophage phenotype in vivo, while the expression levels of genes (e.g., CD206, CD86, Arg-1, iNOS) are commonly used to determine macrophage M1/M2 polarization state in the pathological models [9,12]. In vitro and in vivo studies have demonstrated that the shift of macrophage from M1 to M2 polarity is beneficial for the prevention and treatment of AS [13,14,15].

Asperlin is a fungal natural product which was firstly isolated from marine-derived fungus *Aspergillus* sp. SF-5044 [16,17]. Previous investigations have confirmed its antifungal and anti-inflammatory activities in vitro [17]. Asperlin can inhibit the expression of iNOS and cyclooxygenase (COX)-2, reduce the production of NO and prostaglandin (PG) E2 production, and decrease TNF-α and IL-1β in macrophages [17]. These findings indicated that asperlin showed anti-inflammatory effects in macrophages. However, whether asperlin can exert anti-inflammatory activity in vivo and whether its anti-inflammatory effects are beneficial for the prevention of AS plaque formation remain largely unknown.

In the present study, we isolated asperlin from a marine *Aspergillus versicolor* LZD4403 fungus. The inhibitive effects of asperlin on LPS-evoked foam cell formation and M1/M1 polarization were investigated in RAW264.7 macrophages. Moreover, the anti-inflammatory and atheropreventive activities of asperlin were further confirmed in high-fat diet (HFD)-fed ApoE^−/−^ mice. 

## 2. Results and Discussion

### 2.1. Structural Characterization of Asperlin

**Figure 1:**
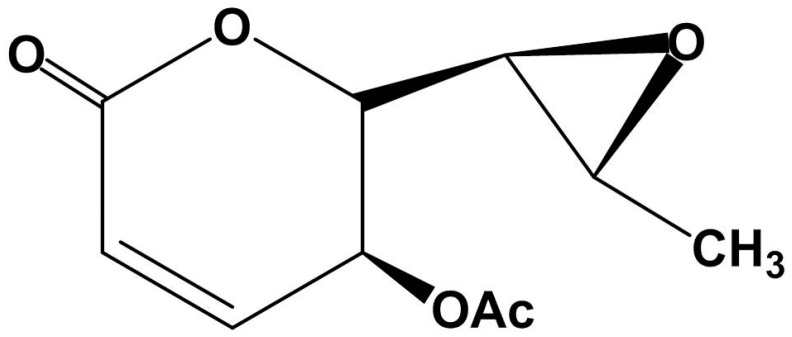
Structure of asperlin.

Chemical examination of a fermentation rice of a marine *Aspergillus versicolor* LZD4403 fungus resulted in the isolation of one known polyketide, termed Asperlin (Figure 1), which was previously isolated from *Aspergillus* sp. IFM51759 fungus [16]. Its structure was elucidated on the basis of extensive nuclear magnetic resonance (NMR) and mass spectroscopic analyses in association with chemical conversion (Table 1).

### 2.2. Asperlin Suppresses LPS-Induced Foam Cell Formation in RAW264.7 Macrophages

**Figure 2:**
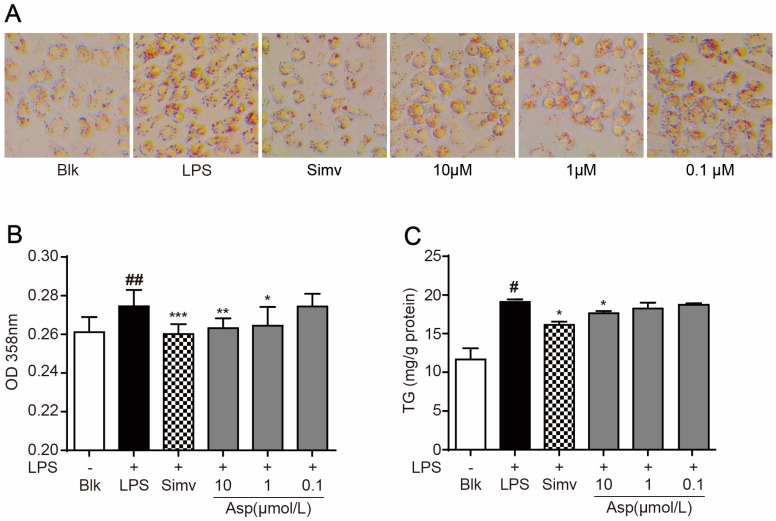
Asperlin suppressed lipopolysaccharide (LPS)-induced foam cell formation in RAW264.7 macrophages. (**A**) Typical images. (**B**) OD 358 nm after oil-red O staining (*n* = 8). (**C**) Intracellular levels of triglyceride (TG) (*n* = 6). Data are shown as means ± SD. ^#^ *p* < 0.05, ^##^ *p* < 0.01, LPS group vs. Blank group; * *p* < 0.05, ** *p* < 0.01, *** *p* < 0.001, test groups vs. LPS group. Blk: blank; Sim: simvastatin; Asp: asperlin.

**Figure 3:**
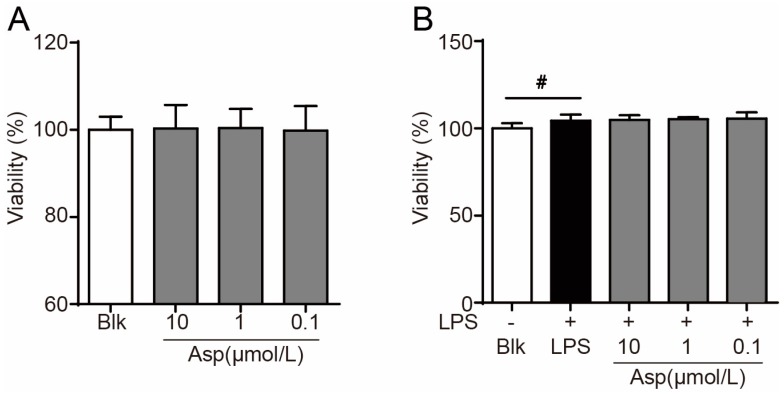
Asperlin showed no influence on cell viability of RAW264.7 macrophages. (**A**) Normal conditions. (**B**) LPS-elicited conditions. Data are shown as means ± SD (*n* = 8). ^#^ *p* < 0.05, LPS group vs. Blank group.

Macrophage-derived foam cell formation plays a crucial role in the development of atherosclerosis [2]. It has been broadly reported that the inflammation-inducing substance LPS can potently promote foam cell formation through suppression of macrophage cholesterol efflux [18,19]. As shown in Figure 2, supplementation of LPS dramatically induced foam cell formation in RAW264.7 macrophages as revealed by oil-red O staining (Figure 2A,B) and intracellular accumulation of triglycerides (Figure 2C), which is in good accordance with previous reports. Treatment with asperlin (1–10 μM) significantly suppressed LPS-induced foam cell formation (Figure 2), with an efficacy comparable to simvastatin, a popular anti-atherosclerosis drug. MTS assay indicated that asperlin showed no influence on the cell viability of RAW264.7 macrophages under normal and LPS-treated conditions (Figure 3), suggesting that the inhibitive effect of asperlin on LPS-induced foam cell formation was not due to cytotoxicity.

### 2.3. Asperlin Stimulates Cholesterol Efflux in LPS-Treated RAW264.7 Macrophages

**Figure 4:**
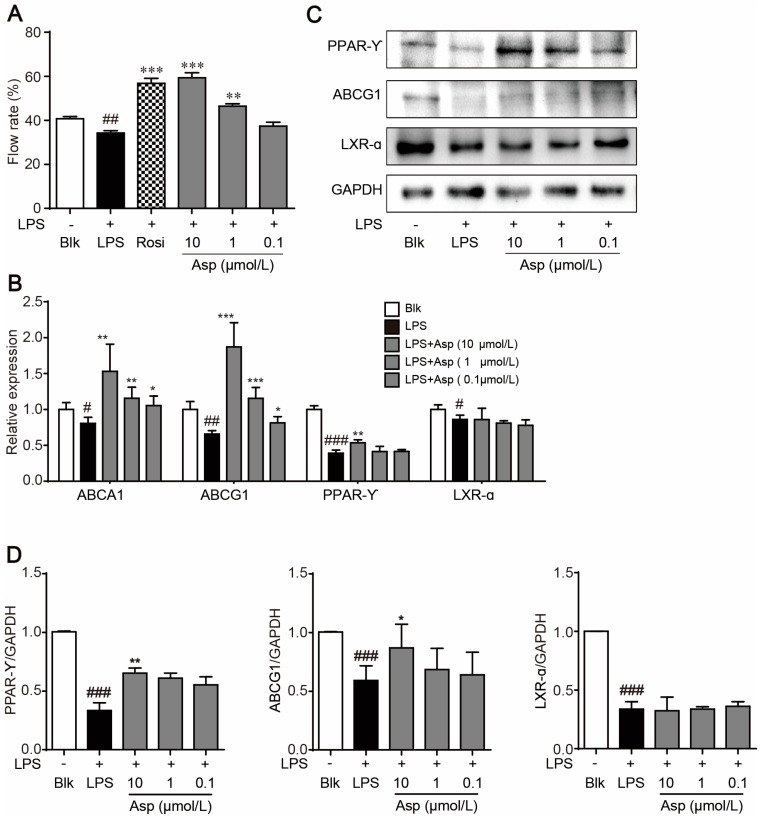
Asperlin stimulated cholesterol efflux in LPS-treated RAW264.7 macrophages. (**A**) Cholesterol efflux rate. (**B**) Realtime PCR of cholesterol efflux-regulating genes. (**C**) Western blotting of cholesterol efflux-regulating proteins. (**D**) Quantitative analysis of the western blots grey. Data are shown as means ± SD. ^#^ *p* < 0.05, ^##^ *p* < 0.01, ^###^ *p* < 0.001, LPS group vs. Blank group; * *p* < 0.05, ** *p* < 0.01, *** *p* < 0.001, test groups vs. LPS group. Blk: blank; Rosi: rosiglitazone; Asp: asperlin.

Stimulating cholesterol efflux is an efficient approach to prevent macrophage foam cell formation as well as atherosclerotic plaque occurrence [20,21]. PPARγ, LXRα, ABCA1 and ABCG1 are key regulators that are critically involved in macrophage cholesterol efflux [21]. Treatment with LPS significantly decreased the cholesterol efflux rate as compared to the vehicle control while addition of asperlin (1–10 μM) substantially reversed the inhibitive effect of LPS (Figure 4A). At the same time, the decreased expression of cholesterol efflux regulators, PPARγ, ABCA1 and ABCG1, by LPS was significantly reversed by asperlin (10 μM) at mRNA levels and PPARγ and ABCG1 at protein levels (Figure 4B,C). These results suggested that asperlin can prevent LPS-evoked foam cell formation through increasing cholesterol efflux.

### 2.4. Asperlin Regulates Macrophage Polarization

**Figure 5:**
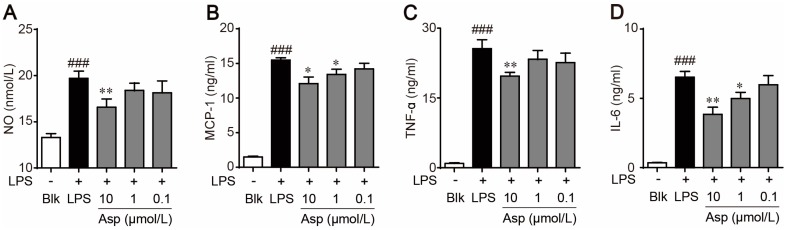
Asperlin inhibited LPS-elicited production of inflammatory factor in RAW264.7 macrophages. (**A**) Nitrite (NO). (**B**) Interleukin-6 (IL-6). (**C**) Tumor necrosis factor α (TNF-α). (**D**) Monocyte chemotactic protein-1 (MCP-1). Data are shown as means ± SD. ^##^ *p* < 0.01, ^###^ *p* < 0.001, LPS group vs. Blank group; * *p* < 0.05, ** *p* < 0.01, *** *p*< 0.001, test groups vs. LPS group. Blk: blank; Asp: asperlin.

**Figure 6:**
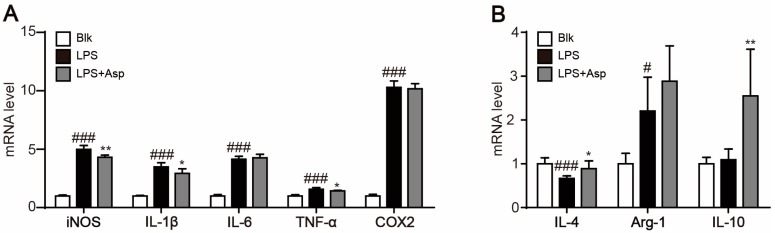
Asperlin regulated macrophage polarization. The transcriptional levels of M1 (**A**) and M2 (**B**) marker genes were determined after treatment with asperlin (10 μM) for 24 h. Data are shown as means ± SD. ^#^ *p* < 0.05, ^###^ *p* < 0.001, LPS group vs. Blank group; * *p* < 0.05, ** *p* < 0.01, test groups vs. LPS group. LPS group. Blk: blank; Asp: asperlin.

The shift in M1/M2 macrophages polarization has been implicated in the anti-inflammatory and atherosclerotic protective effects of many natural products [22]. Treatment with asperlin significantly decreased LPS-elicited production of pro-inflammatory factors such as NO, IL-6, TNF-α and MCP-1 in RAW264.7 macrophages, indicating potent anti-inflammatory activities (Figure 5). To investigate whether asperlin inhibited LPS-evoked inflammation through regulating macrophages polarization, the mRNA levels of typical markers for M1 macrophages (iNOS, IL-1β, TNFα, COX-2 and IL-6) and markers for M2 macrophages (Arg-1, IL-10 and IL-4) were quantified by realtime polymerase chain reaction (PCR). As shown in Figure 6, the expression levels of M1 markers iNOS, IL-1β and TNFα were significantly decreased while M2 markers IL-10 and IL-4 were markedly increased by asperlin. These results suggested that asperlin may inhibit inflammation through shifting macrophages polarization from M1 to M2.

### 2.5. Asperlin Does Not Inhibit Foam Cell Formation Induced by oxLDL in RAW264.7 Macrophages

**Figure 7:**
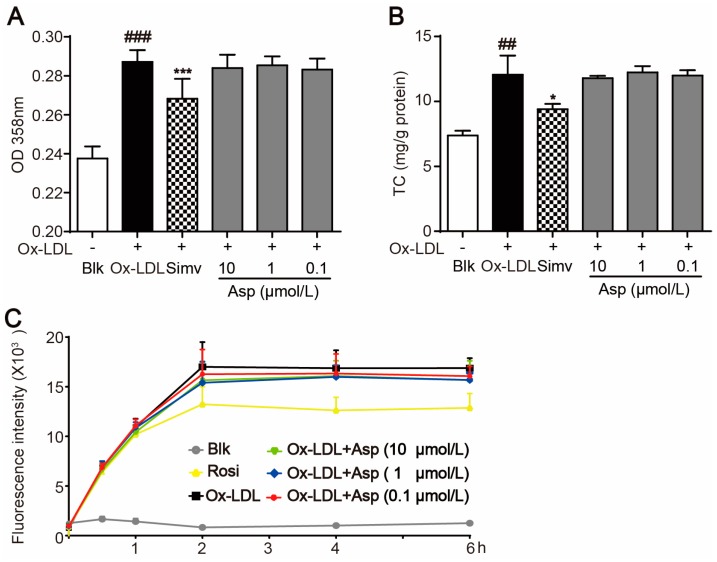
Asperlin did not inhibit foam cell formation induced by ox-LDL in RAW264.7 macrophages. (**A**) OD 358nm after oil-red O staining. (**B**) Intracellular levels of cholesterol (TC). (**C**) Time-dependent cholesterol uptake. Data are shown as means ± SD. ^##^ *p* < 0.01, ^###^ *p* < 0.001, ox-LDL group vs. Blank group; * *p* < 0.05, *** *p* < 0.001, test groups vs. ox-LDL group. Blk: blank; Rosi: rosiglitazone; Asp: asperlin.

Besides inflammatory factors, modified LDL is another key promoter for foam cell formation. To investigate the effect of asperlin on modified LDL-induced foam cell formation, oxidized LDL (oxLDL) was used to establish macrophage foam cells. In contrast to its potent inhibitive activity in LPS-evoked foam cells, asperlin exhibited no influence on oxLDL-induced macrophage foam cell formation, as revealed by oil-red O staining (Figure 7A), intracellular cholesterol accumulation (Figure 7B) and time-dependent cholesterol uptake (Figure 7C). These results suggested that asperlin suppresses foam cell formation rather through inhibiting LPS-induced inflammation than decreasing lipid accumulation.

### 2.6. Asperlin Prevents HFD-Induced Atherosclerosis in ApoE^−/−^ Mice

**Figure 8:**
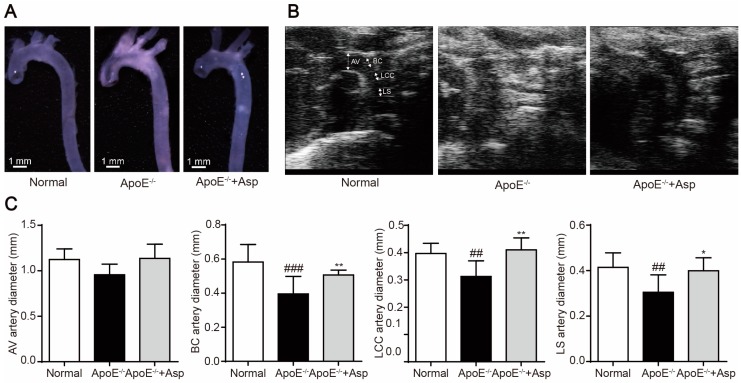
Treatment with Asperlin for 12 weeks prevents high-fat diet (HFD)-induced atherosclerosis in apoE^−/−^ mice. (**A**) Atherosclerotic plaques (arrows) in aortic arches. (**B**) In vivo ultrasound. (**C**) Measurement of the diameter of the ascending aorta 2 cm above the aortic valve (AV, arrow), the brachiocephalic (BC) artery, left common carotid (LCC) artery and left subclavian (LS) artery. Data are shown as means ± SD. ^#^ *p* < 0.05, ^##^ *p* < 0.01, ^###^ *p* < 0.001, apoE^−/−^ group vs. normal group; * *p* < 0.05, ** *p* < 0.01, test groups vs. apoE^−/−^ group. Asp: asperlin.

To confirm the preventive effect of asperlin on atherosclerosis development, we fed ApoE^−/−^ mice with a cholesterol-rich diet for 12 weeks to establish an atherosclerotic animal model and investigate the atheropreventive effect of asperlin in vivo. Treatment with asperlin affected neither food intake nor body weight (data not shown), but remarkably suppressed atherosclerotic plaque formation in aorta. The artery morphology showed that treatment with asperlin (80 mg/kg) for 12 weeks remarkably reduced the white atherosclerotic lesions as compared to the vehicle control (Figure 8A). The vascular lumen diameter was measured by high frequency ultrasound [23,24], which displayed that Asperlin (80 mg/kg) significantly reduced vascular wall thickness of the ascending aorta at 2 cm above the aortic valve (AV, arrow), the brachiocephalic (BC) artery, left common carotid (LCC) artery and left subclavian (LS) artery (Figure 8B,C), suggesting that Asperlin effectively inhibited aortic dilatation in ApoE^−/−^ mice. 

**Figure 9:**
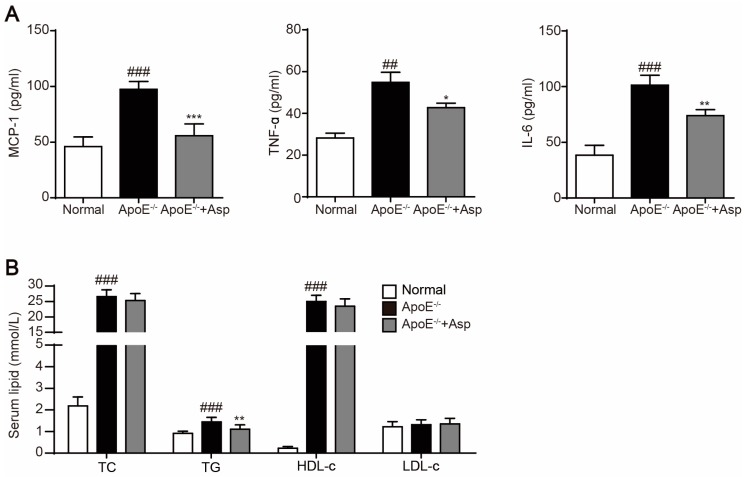
Asperlin markedly ameliorates inflammation but shows weak influence on hyperlipidemia in HFD-induced atherosclerotic ApoE^−/−^ mice. (**A**) Serum levels of interleukin-6 (IL-6), tumor necrosis factor α (TNF-α), monocyte chemotactic protein-1 (MCP-1). (**B**) Serum levels of lipids. Data are shown as means ± SD. ^##^ *p* < 0.01, ^###^ *p* < 0.001, apoE^−/−^ group vs. normal group; * *p* < 0.05, ** *p* < 0.01, *** *p* < 0.001, test groups vs. apoE^−/−^ group. Asp: asperlin.

We also measured the serum concentration of TC, TG, LDL-c and HDL-c as well as serum levels of pro-inflammatory factors MCP-1, TNF-α and IL-6. As shown in Figure 9, asperlin significantly decreased serum levels of MCP-1, TNF-α and IL-6 in ApoE^−/−^ atherosclerotic mice, exhibiting potent anti-inflammatory effect (Figure 9A). However, treatment with asperlin for 12 weeks showed minimal impact on blood lipids except TG (Figure 9B). These results indicated that asperlin is adequate to prevent atherosclerosis in vivo. It may exert atheroprotective function through suppressing inflammation rather than ameliorating dyslipidemia.

## 3. Experimental Section

### 3.1. Materials and Reagents

25-[*N*-[(7-nitrobenz-2-oxa-1,3-diazol-4-yl)-methyl]amino]-27-norcholesterol (25-NBD cholesterol), lipopolysaccharide (LPS), simvastatin, oil-red O, 3-(4,5-dimethylthiazol-2-yl)-5-(3-carboxymethoxyphenyl)-2-(4-sulfophenyl)-2H-tetrazolium (MTS) and Dulbecco’s modified Eagle’s medium (DMEM) were procured from Sigma-Aldrich (St. Louis, MO, USA). The oxidized low-density lipoprotein (oxLDL) was from Xiesheng Biotechnologies, Inc. (Beijing, China). The kits for triglycerides (TG), total cholesterol (TC), LDL-c, HDL-c, interleukin-6 (IL-6), tumor necrosis factor a (TNF-α) and monocyte chemotactic protein-1 (MCP-1) were purchased from Jian Cheng Biotechnology Company (Nanjing, China). Total RNA extraction reagent, PrimeScript RT reagent kit and SYBR-Green PCR kit were purchased from Transgene Biotech, Inc. (Beijing, China). All the antibodies used in this study were acquired from Cell Signaling Technology (CST) (Beverly, MA, USA).

### 3.2. Isolation and Identification of Asperlin

#### 3.2.1. Fungal Material

Fungal strain *Aspergillus versicolor* LZD4403 was isolated from gorgonian (*Pseudopterogorgia* sp.), which was collected from Leizhou Island in Guangdong Province of China, in June 2015. The fungus was identified by comparing the morphological character and ITS sequence (GenBank number KY744355) with those of standard records. All experiments and observations were repeated at least twice leading to the identification of the strain as *A. versicolor*. This strain (LZD4403) was deposited at the State Key Laboratory of Natural and Biomimetic Drugs, Peking University, China.

#### 3.2.2. Fermentation

Fermentation of the strain was initiated in 500 mL sized Erlenmeyer flasks, each preloaded with 80 g of rice and 100 mL of sterilized artificial seawater. The seed was prepared by inoculating activated fungal cakes from an agar Petri dish into 200 mL of potato dextrose broth medium. Approximately 20 mL aliquots of the inoculum were then transferred to fermentation medium and further incubated for 30 days at 28 °C statically.

#### 3.2.3. Extraction and Isolation

The rice fermentation was extracted three times with EtOAc, and then was concentrated under in vacuo (32 °C) to give an extract, which was subsequently subjected to silica gel vacuum liquid chromatography, eluting with petroleum ether-ethyl acetate (PE-EtOAc) (from 50:1 to 0:100, gradient), to obtain 7 fractions (F1–F7, 27.88 g). F3 (5.60 g) was subjected to a Sigel CC column (160–200 mesh) eluting with PE:EtOAc = 7:1 to yield three portions (P1–P3). A gel filtration over Sephadex LH-20 of P2 (5.04 g) eluting with MeOH was employed to afford five portions (P2_1–5_). P2_2_ (4.65 g) was further separated by Sephadex LH-20 eluting with MeOH and recrystallization to obtain asperlin (4.50 g).

#### 3.2.4. Structural Identification

Optical rotations were measured on a Rudolph IV Autopol automatic polarimeter. IR spectra were determined on a Thermo Nicolet Nexus 470 FT-IR spectometer. 1D and 2D NMR spectra were recorded on Bruker Advance 500 NMR spectrometer (500 MHz for ^1^H and 125 MHz for ^13^C, respectively). Chemical shifts are expressed in *δ* (ppm) referenced to the solvent peaks at *δ*_H_ 2.50 and *δ*_C_ 39.8 for DMSO-*d*_6_, and coupling constants are in Hz. ESIMS spectra were obtained from a Thermo Scientific LTQ Orbitrap XL instrument (Thermo Fisher Scientific Co. Ltd., Bremen, Germany). Silica gel (160–200 and 200–300 mesh) for TLC was obtained from Qingdao Marine Chemistry Co. Ltd. (Qingdao, China). Sephadex LH-20 (18–110 μm) was obtained from YMC Co. Ltd. (Kyoto, Japan) and Amersham Pharmacia Biotech AB (Uppsala, Sweden). Precoated silica gel plates (Kieselgel60 F254, 0.25 mm) (Merck, Germany) were used for TLC analysis.

### 3.3. Cell Culture

RAW264.7 cells were originated from the American Type Culture Collection (ATCC) (Manassas, VA, USA) and obtained from the Peking Union Medical College (Beijing, China). RAW264.7 cells, were maintained in DMEM containing 10% FBS at 37 °C and 5% CO_2_. When grown to 70–80% confluence, cells were kept in serum-free DMEM and incubated with the different concentrations of Asperlin (10, 1, 0.1 μmol/L) or with simvastatin (10 μmol/L) in DMEM containing LPS (10 ng/mL) for 24 h. The blank group was incubated with serum-free DMEM alone. Asperlin was dissolved in dimethyl Sulphoxide (DMSO) and the control group was given equal volume of DMSO.

### 3.4. Cell Viability Assay

Cell viability was evaluated by MTS assay. RAW264.7 cells were maintained in DMEM with 10% FBS, and penicillin/streptomycin (100 μg/mL) and incubated at 37 °C and 5% CO_2_. The cells were seeded into 96-well plates at the concentration of 1 × 10^5^ cells/mL and cultured for 24 h. Then cells were supplemented with different concentrations of asperlin (10, 1, 0.1 μmol/L) with or without LPS (10 ng/mL) for 24 h. The MTS (20 μL) solution was added to each well and incubated for 4 h. The optical density was assessed at 490 nm by a Tecan Infinite M1000Pro Microplate Reader (TECAN Group Ltd., Shanghai, China) and the relative cell viability was calculated.

### 3.5. Oil-Red O Staining

The cells with 70–80% confluence in 96 well plates were incubated with serum-free DMEM + LPS (10 ng/mL) and different concentrations of Asperlin (10, 1, 0.1 μmol/L) or the positive control simvastatin (10 μmol/L) for 24 h. Cells were then fixed with 4% *w*/*v* paraformal dehyde (30 min, room temperature) and stained with 0.5% filtered oil-red O solution (15 min, room temperature). The staining was evaluated by a Tecan Infinite M1000Pro Microplate Reader and spectrophotometry at 358 nm.

### 3.6. Intracellular TG Quantification

RAW264.7 cells were maintained in DMEM medium supplemented with 10% FBS and penicillin/streptomycin (100 μg/mL). The cells with 70–80% confluence in 6 well plates were treated with serum-free DMEM + LPS (10 ng/mL) and different concentrations of Asperlin (10, 1, 0.1 μmol/L) or the positive control simvastatin (10 μmol/L) for 24 h. Subsequently, the cells were subjected to TG quantification as described previously [25,26]. Each experiment was repeated in triplicate.

### 3.7. Cholesterol Efflux Assay

RAW264.7 cells were equilibrated with 25-NBD cholesterol (5 μg/mL) for 12 h. The 25-NBD cholesterol-labeled cells were washed with PBS twice and then incubated in serum-free DMEM containing HDL (50 μg/mL) + LPS (10 ng/mL) and different concentrations of Asperlin (10, 1, 0.1 μmol/L) or the positive control rosiglitazone (10 μmol/L) for 6 h. Fluorescence-labeled cholesterol that released from the cells into the medium was measured with a Tecan Infinite M1000Pro Microplate Reader. Cholesterol efflux was expressed as a percentage of fluorescence in the medium relative to the total amount of fluorescence detected in the cells and the medium. Each experiment was repeated in triplicate.

### 3.8. Realtime Quantitative PCR

Total RNA extraction, cDNA synthesis and quantitative PCR assays were performed as described previously [21]. At least three independent biological replicates were performed to verify the reproducibility of the data. The gene-specific primers used for quantitative PCR are listed in Table 2.

### 3.9. Western Blotting

Whole cell protein extraction and Western blotting were per formed as previously described [27]. Briefly, cells were lysed on ice by incubation in cold RIPA lysis buffer for 30 min. Proteins were obtained by centrifugation, resolved on 8.5% polyacrylamide gels, and subsequently transferred onto nitrocellulose membranes. Immunoblotting was performed with respective antibodies (1:1000). Following incubation with horseradish peroxidase-conjugated secondary antibody, proteins were detected with ECL plus kits (Amersham, Piscataway, NJ, USA). The primary antibodies used in the experiment were purchased from Abcam (Cambridge, MA, USA). The catalog numbers were as follows, PPARγ (ab59256), LXRα (ab3585), ABCG1 (ab52617) and GAPDAH (ab22555). At least three independent western blot experiments were performed for protein.

### 3.10. Determination of NO Production in RAW264.7 Cells

Cells were plated at 2 × 10^5^ cells/well in 96-well plates and maintained for 24 h. The cells were incubated with Asperlin (10, 1, 0.1 μmol/L) and exposed to LPS (10 μg/mL) for 24 h. The blank control cells were treated with DMEM only. LPS-induced NO production was determined by analyzing the NO level using Griess reagent (Beyotime Inc., Beijing, China), and the absorbance at 540 nm was measured using a Tecan Infinite M1000Pro Microplate Reader (TECAN Group Ltd., Shanghai, China).

### 3.11. Measurement of MCP-1, IL-6 and TNFα in RAW264.7 Cells

RAW264.7 cells were cultured in 24-well plates (2 × 10^5^ cells/mL), incubated with different concentrations of Asperlin (10, 1 μmol/L) and exposed to LPS (10 μg/mL) for 24 h. The blank control cells were treated with DMEM only. Cell culture supernatants were collected immediately after treatment and centrifuged at 13,000× *g* for 2 min to detect cytokines. The levels of TNFα, IL-6 and TNFα were determined with respective kits according to the manufacturer’s instructions.

### 3.12. Animal Experiment

All animal experiments were approved by the Medical Ethics Committee of Peking Union Medical College and were in accordance with the National Institutes of Health regulations for the care and use of animals in research. All efforts were made to minimize suffering.

Male C57BL/6 J and C57BL/6 J ApoE^−/−^ mice (6–8 weeks old), weighting 20–25 g, were purchased from Vital River Laboratory Animal Technology Co., Ltd. (Beijing, China). The animals were kept in a humidity-controlled room on a 12 h light/12 h dark cycle and adapted to the new environment for one week. The ApoE^−/−^ mice were then divided randomly into two groups with ten animals in each group and fed the Western Diets (21.0% anhydrous milk fat, 34% sucrose, 20.0% protein and 0.15% cholesterol) for 12 weeks. The C57BL/6 J mice were fed with standard diet. The negative control group (ApoE^−/−^ group) was given equal volume of distilled water while the ApoE^−/−^ + Asp group was administrated by oral gavage with Asperlin (80 mg/kg) once a day. Asperlin was suspended in 0.5% sodium carboxymethyl cellulose (CMC-Na). The control mice were given equal volume of 0.5% CMC-Na. At the end of the study, after the animals were fasted overnight, blood samples were collected for estimation of plasma levels of lipids and inflammatory factors (MCP-1, IL-6 and TNFα) by kits. Animals were then euthanized, and the entire aorta were collected and photographed with a microscope (SZ61, Olympus, Japan). The lesion areas were quantified with ImageJ software.

### 3.13. In Vivo Ultrasound

After treatment with Asperlin for 11 weeks, mice were anesthetized with inhaled 1–2% isoflurane titrated to a heart rate of 470–500 beats per minute and shaved. The ascending aorta was visualized in one plane from the aortic valve to the transverse aorta in a parasternal long axis view using a 40 MHz high frequency Visual Sonics Vevo 660 ultrasound machine. The diameter of the ascending aorta 2 cm above the aortic valve, brachiocephalic artery, left common carotid artery and left subclavian artery were measured by the leading edge method.

### 3.14. Statistical Analysis

Data are shown as the mean ± SD. One-way ANOVA was used to determine significant differences among groups, after which the modified Student’s *t*-test with the Bonferroni correction was used for comparison between individual groups. *p* < 0.05 was considered statistically significant. SPSS 17.0 for Windows (SPSS, Chicago, IL, USA) was used for statistical analysis.

## 4. Conclusions

The present work demonstrated asperlin, a known polyketide isolated from marine *Aspergillus versicolor* LZD4403 fungus, as an effective agent that inhibited LPS-induced foam cell formation in RAW264.7 macrophages and prevented atherosclerosis development in ApoE^−/−^ mice. Mechanistic investigation revealed that asperlin promoted cholesterol efflux through upregulation of the PPARγ-ABCA1/G1 pathway and shifted macrophages polarization from M1 to M2 macrophages. The suppressive effect of asperlin on macrophages foam cell formation and atherosclerosis development was mediated by inhibiting inflammation rather than ameliorating dyslipidemia. These findings support the idea that marine-derived asperlin is a promising candidate for the prevention and treatment of athersclerosis.

## Figures and Tables

**Table 1:** ^1^H NMR (500 MHz) and ^13^C NMR (125 MHz) data in dimethyl Sulphoxide (DMSO)-*d*_6_.

Position	*δ*_H_ (mult., *J* in Hz)	*δ*_C_	Position	*δ*_H_ (mult., *J* in Hz)	*δ*_C_
**1**			**7**	3.02, *dd* (6.5, 2.0)	55.4
**2**		162.2	**8**	3.11, *dd* (6.5, 2.0)	52.8
**3**	6.25, *d* (12.0)	124.9	**9**	1.28, *d* (6.5)	17.3
**4**	7.09, *dd* (12.0, 7.5)	141.5	**10**		170.0
**5**	5.39, *dd* (7.5, 3.5)	62.0	**11**	2.10, *s*	20.8
**6**	4.55, *dd* (6.5, 3.5)	77.5			

**Table 2:** The primers used in this work.

Name	Forward (5′-3′)	Reverse (5′-3′)
ABCA1	GCGGACCTCCTGGGTGTT	CAAGAATCTCCGGGCTTTAGG
ABCG1	AAGGCCTACTACCTGGCAAAGA	GCAGTAGGCCACAGGGAACA
PPARγ	CATTCTGGCCCACCAACTTC	TCAAAGGAATGCGAGTGGTCTT
LXRα	CCTTCCTCAAGGACTTCAGTTACAA	CATGGCTCTGGAGAACTCAAAGAT
iNOS	GCATCCCAAGTACGAGTGGT	CCATGATGGTCACATTCTGC
IL-1β	GGAGAAGCTGTGGCAGCTA	GCTGATGTACCAGTTGGGGA
IL-6	CCGGAGAGGAGACTTCACAG	TGGTCTTGGTCCTTAGCCAC
TNF-α	GACCCTCACACTCAGATCAT	TTGAAGAGAACCTGGGAGTA
COX-2	GCTGTACAAGCAGTGGCAAA	GTCTGGAGTGGGAGGCACT
IL-4	GGTCTCAACCCCCAGCTAGT	GCCGATGATCTCTCTCAAGTGAT
Arg-1	TGTCCCTAATGACAGCTCCTT	GCATCCACCCAAATGACACAT
IL-10	CTTACTGACTGGCATGAGGATCA	GCAGCTCTAGGAGCATGTGG
β-actin	GGCTGTATTCCCCTCCATCG	CCAGTTGGTAACAATGCCATGT
